# Genetic diversity analysis and core collection construction of tillering onion (*Allium cepa* L. var. *aggregatum* G. Don) based on phenotypic traits and SNP markers

**DOI:** 10.3389/fpls.2026.1969213

**Published:** 2026-10-30

**Authors:** Yuzhu Han, Huiying Wang, Jing Zhao, Zhijun Han, Qunzhu Cheng

**Affiliations:** 1 College of Agriculture, JiLin Agricultural Science and Technology College, Jilin, China; 2 College of Horticulture, Jilin Agricultural University, Changchun, Jilin, China; 3 Baicheng Academy of Agricultural Sciences, Baicheng, Jilin, China; 4 Institute of Economic Plants, Jilin Academy of Agricultural Sciences, Changchun, Jilin, China; 5 Longyao County Agriculture and Rural Affairs Bureau, Xingtai, Hebei, China

**Keywords:** core collection, fingerprint, genetic diversity, phenotypic traits, SNP markers, tillering onion

## Abstract

**Introduction:**

Tillering onion (*Allium cepa* L. var. *aggregatum* G. Don) is a specialty vegetable widely grown in China, yet its germplasm resources remain poorly characterized at the genetic level. The aim of this study was to characterize the phenotypic and molecular diversity of tillering onion germplasm and to construct a representative core collection.

**Methods:**

We evaluated 87 tillering onion accessions collected from six Chinese provinces using 29 agronomic traits and single-nucleotide polymorphism (SNP) markers developed via specific-locus amplified fragment sequencing (SLAF-seq). Phenotypic diversity was assessed by coefficients of variation, genetic diversity indices, correlation analysis, principal component analysis (PCA), stepwise regression, and UPGMA clustering. Molecular diversity was characterized through population structure analysis and a high-quality SNP fingerprint. A core collection was constructed by integrating phenotype-based (QGA) and genotype-based (Core Hunter) approaches and was validated using mean difference percentage (MD%), coincidence rate of range (CR%), and variable rate of coefficient of variation (VR%).

**Results:**

Phenotypic coefficients of variation ranged from 14.27% to 65.41%, and genetic diversity indices ranged from 0.514 to 2.234. Correlation analysis identified 137 significantly correlated trait pairs (*p* < 0.05), including 100 at *p* < 0.01. PCA extracted 10 principal components accounting for 79. 31% of cumulative variance, and stepwise regression identified 14 representative traits (R² = 0.971). UPGMA clustering classified the accessions into eight groups. At the molecular level, SLAF-seq yielded 1,209,763 SNP loci, and population structure analysis divided the accessions into two subpopulations, with C1 showing slightly higher diversity (Nei's index = 0.281, PIC = 0.224) than C2. A fingerprint based on 335 high-quality SNP markers showed population-wide observed heterozygosity of 0.379–0.644 (mean 0.572), Nei's diversity index of 0.455–0.500 (mean 0.479), and PIC of 0.352–0.375 (mean 0.364). The core collection comprised nine accessions (M15, M24, M28, M37, M45, M47, M49, M62, M66), representing 10.34% of the total resources, with validation parameters of MD% = 10.0%, CR% = 83.6%, and VR% = 112.2%.

**Discussion:**

These results provide a framework for germplasm conservation, elite accession identification, and parental selection in tillering onion breeding.

## Introduction

Tillering onion (*Allium cepa* L. var. *aggregatum* G. Don), also known as multiplier onion or potato onion, is a biennial herbaceous plant in the family Amaryllidaceae grown widely in northeastern China (Li et al., 2023). The crop is valued for its distinctive tillering habit, stress tolerance, and nutrient-rich bulbs containing flavonoids, soluble sugars, proteins, and other bioactive compounds (Huang et al., 2019). Despite these qualities, tillering onion breeding has relied mainly on selection within local landraces, and the genetic relationships among accessions remain poorly understood. This lack of systematic characterization constrains both germplasm utilization and cultivar development.

Genetic diversity evaluation is fundamental to clarifying the genetic background of germplasm and guiding parental selection (Yang, 2021). Agronomic trait assessment remains the most straightforward approach—intuitive, inexpensive, and directly relevant to breeding goals (Yang et al., 2014)—but it is limited by environmental effects and low resolution among closely related materials. Molecular markers overcome these limitations by detecting variation at the DNA level, offering high stability and abundant polymorphism independent of environmental conditions (Wang et al., 2025).

Among molecular marker systems, single nucleotide polymorphism (SNP) markers are particularly well suited for genetic diversity analysis owing to their abundance, genome-wide distribution, and compatibility with high-throughput genotyping (Davey et al., 2011). Specific-locus amplified fragment sequencing (SLAF-seq) enables cost-effective, large-scale SNP discovery and has been applied to germplasm evaluation in crops such as flax (Gao, 2018) and black goji berry (Yang, 2024). Gao (2018) genotyped 317 flax accessions with SNP markers and developed fingerprints for resource management. Yang (2024) combined phenotypic and SNP data to construct a core collection of black goji berry, demonstrating a strategy applicable to other underutilized crops.

A core collection captures the maximum genetic diversity of a species with the fewest possible accessions (Frankel and Brown, 1983). Since this concept was introduced, it has become a standard tool for efficient germplasm management. Combining phenotypic and molecular data in core collection construction provides a more complete picture of genetic diversity than either data type alone (Yan, 2021).

Research on tillering onion germplasm is still at an early stage. Li et al. (2023) evaluated agronomic and quality traits in 69 accessions; Jin et al. (2013) assessed genetic diversity in 48 accessions using ISSR markers; and Rungis et al. (2021) characterized 110 Baltic onion landraces with SSR markers. None of these studies employed SNP markers or constructed a core collection, leaving the genetic structure of tillering onion germplasm largely unexplored.

In this study, 87 tillering onion accessions from six Chinese provinces were characterized using 29 agronomic traits and SNP markers generated by SLAF-seq. The objectives were to (1) assess phenotypic and molecular genetic diversity, (2) analyze population structure and construct SNP fingerprints, and (3) develop a core collection through integrated phenotypic and genotypic selection. The resulting core collection and molecular resources will facilitate germplasm conservation, elite accession identification, and breeding in tillering onion.

## Materials and methods

### Plant materials

A total of 87 tillering onion germplasm accessions were collected from six provinces in China: 45 from Jilin Province, 21 from Heilongjiang Province, 9 from Guangxi Zhuang Autonomous Region, 7 from Liaoning Province, 3 from Shandong Province, and 2 from Hebei Province (**Table 1**). All materials were morphologically identified and confirmed as tillering onion. The accessions were collected in 2023, and local names follow the collection-site names. The detailed collection sites (village/town level) for each accession are provided in **Table 1**. All accessions are farmer-maintained landraces propagated vegetatively. The identity and uniformity of the accessions were further confirmed at the molecular level: based on the 335 fingerprint markers, no two of the 87 accessions shared an identical genotype profile (**Supplementary Table S1**), and no putative genetic duplicates were detected.

**Table 1:** Sources and accession numbers of 87 tillering onion germplasm resources.

Accession no.	Origin	Accession no.	Origin	Accession no.	Origin
M1	Acheng District, Harbin City, Heilongjiang Province	M30	Kaiyuan City, Liaoning Province	M59	Dongbailing Village, Yangshulin Township, Jilin Province
M2	Wuchang City, Heilongjiang Province	M31	Changtu County, Kaiyuan City, Liaoning Province	M60	Qingfeng Village, Wangshun Township, Jilin Province
M3	Fujing City, Heilongjiang Province	M32	Fushun City, Liaoning Province	M61	Liujiazi Village, Yangshulin Township, Jilin Province
M4	Tieli City, Heilongjiang Province	M33	Lingyuan County, Liaoning Province	M62	Liujiazi Village, Yangshulin Township, Jilin Province
M5	Daqing City, Heilongjiang Province	M34	Jinzhou City, Liaoning Province	M63	Qingfeng Village, Wangshun Township, Jilin Province
M6	Shuibin County, Heilongjiang Provinc	M35	Zangjiakou City, Hebei Province	M64	Qingfeng Village, Wangshun Township, Jilin Province
M7	Yichun City, Heilongjiang Province	M36	Chengde City, Hebei Province	M65	Qingfeng Village, Wangshun Township, Jilin Province
M8	Yi’an County, Heilongjiang Province	M37	Lingshan County, Guangxi Zhuang Autonomous Region	M66	Dongbailing Village, Yangshulin Township, Jilin Province
M9	Keshan County, Heilongjiang Province	M38	Guigang City, Guangxi Zhuang Autonomous Region	M67	Yushu Village, halahai Town, Jilin Province
M10	Qinjin Town, Heilongjiang Province	M39	Liuzhou City, Guangxi Zhuang Autonomous Region	M68	Yushu Village, halahai Town, Jilin Province
M11	Yichun City, Heilongjiang Province	M40	Sandu Town, Guangxi Zhuang Autonomous Region	M69	Jinjiazi Village, halahai Town, Jilin Province
M12	Xuma Village, Heilongjiang Province	M41	Wulajie Town, Jilin Province	M70	Qingfeng Village, Wangshun Township, Jilin Province
M13	Aaling Town, Jilin Province	M42	Rendong Town, Yulin City, Guangxi Zhuang Autonomous Region	M71	halahai Town, Jilin Province
M14	Yongfa Township, Gongzhuling City, Jilin Province	M43	Bobai County, Yulin City, Guangxi Zhuang Autonomous Region	M72	Qingfeng Village, Wangshun Township, Jilin Province
M15	Yushu City, Jilin Province	M44	Nanning City, Guangxi Zhuang Autonomous Region	M73	Jinjiazi Village, halahai Town, Jilin Province
M16	Siping City, Jilin Province	M45	Pumiao Town, Nanning City, Guangxi Zhuang Autonomous Region	M74	Qingfeng Village, Wangshun Township, Jilin Province
M17	Shuangliao City, Jilin Province	M46	Qinzhou City, Guangxi Zhuang Autonomous Region	M75	Qingfeng Village, Wangshun Township, Jilin Province
M18	Baoqing County, Heilongjiang Province	M47	Wulajie Town, Jilin Province	M76	Dongbailing Village, Yangshulin Township, Jilin Province
M19	Shuangyashan City, Heilongjiang Province	M48	Bolinchengzi Town, Gongzhuling City, Jilin Province	M77	Qingfeng Village, Wangshun Township, Jilin Province
M20	Jiaxinzi Town, Heilongjiang Province	M49	Qiqihaer City, Heilongjiang Province	M78	Qingfeng Village, Wangshun Township, Jilin Province
M21	Hailun City, Heilongjiang Province	M50	Zhaodong City, Heilongjiang Province	M79	Wulajie Town, Jilin Province
M22	Qingyuan Town, Heilongjiang Province	M51	Qinguo County, Songyuan City, Jilin Province	M80	Wulajie Town, Jilin Province
M23	Zhaodong City, Heilongjiang Province	M52	Qingfeng Village, Wangshun Township, Jilin Province	M81	Sanhe Township, Dongfeng County, Jilin Province
M24	Liaoyuan City, Jilin Province	M53	Yangshulin Township, Jilin Province	M82	anhe Township, Dongfeng County, Jilin Province
M25	Jinning City, Shandong Province	M54	Yangshulin Township, Jilin Province	M83	Changchun City, Jilin Province
M26	Tai’an City, Shandong Province	M55	Chaigang County, Jilin Province	M84	Changchun City,Jilin Province
M27	Liaocheng City, Shandong Province	M56	Chaigang County, Jilin Province	M85	Nong’an City, Jilin Province
M28	Tieling City, Liaoning Province	M57	Qingfeng Village, Wangshun Township, Jilin Province	M86	Gannan Town, Qiqiha’er City, Heilongjiang Province
M29	Tieling City, Liaoning Province	M58	Yangshulin Township, Jilin Province	M87	Songyuan City, Jilin Province

### Phenotypic trait measurement and data analysis

#### Field experimental design

The 87 accessions were sown on April 9, 2024, at the experimental field of Jilin Agricultural Science and Technology University (126.46°E, 43.95°N) and harvested in early July. A randomized complete block design with three replicates was used. Each plot was 2 m in row length with 15 cm plant spacing and a plot area of 0.72 m², using zigzag planting with conventional field management.

#### Trait measurement methods

Twenty-nine agronomic traits were measured following the “Descriptors and Data Standards for Onion Germplasm Resources” (Zhan and Li, 2008), including 19 quantitative traits (plant height, pseudostem height, pseudostem diameter, leaf number, tube leaf length, tube leaf transverse diameter, leaf spread, tillering number, membranous scale leaf number, flesh scale number, flesh scale stripe number, flesh scale thickness, bulb neck diameter, bulblet number, disc stem diameter, bulb longitudinal diameter, bulb transverse diameter, bulb weight, yield per plant), 5 qualitative traits (leaf color, leaf waxiness, plant morphology, pseudostem skin color, scale leaf color), and 5 quality traits (soluble solids content, soluble sugar content, soluble protein content, dry matter content, total flavonoid yield). For quantitative traits, 10 plants with uniform growth were randomly selected from each plot and measured using the fixed-plant method. Qualitative traits were scored according to grading standards. Soluble solids content was measured with an Abbe refractometer; soluble sugar and soluble protein contents were determined by the anthrone colorimetric method and Coomassie Brilliant Blue G-250 method, respectively (Wang, 2006); dry matter content was measured by oven-drying; and total flavonoid yield was determined by ultrasound-assisted extraction (Huang et al., 2019).

#### Data analysis

Descriptive statistics (mean, standard deviation, and coefficient of variation) and the Shannon–Wiener diversity index (H′) were calculated from the raw trait data. Before principal component analysis and cluster analysis, all quantitative traits were standardized to a mean of 0 and a standard deviation of 1. Pearson correlation analysis was used to test correlations among traits. Principal component analysis was performed using the prcomp function in R. Cluster analysis was conducted using squared Euclidean distance and the unweighted pair group method with arithmetic mean (UPGMA), implemented with the hclust function in R. For the field experiment, the ten plants sampled per plot were treated as subsamples and averaged within each plot, so that the plot served as the experimental unit. Accession means for each trait were estimated using a linear mixed model with block as a random effect, and the adjusted means (BLUPs) were used as inputs for PCA and cluster analysis. For quantitative traits, the Shannon–Wiener diversity index was calculated by dividing each trait into k = 5 classes, computing the relative frequency p_i_ of each class, and applying H′ = −Σ p_i_ ln(p_i_).

### SNP marker analysis

#### Genomic DNA extraction and SLAF-seq library construction

Healthy leaves were collected during the vigorous leaf growth stage, and genomic DNA was extracted using a modified CTAB method. After DNA quality was verified by 1% agarose gel electrophoresis and microvolume spectrophotometry, SLAF-seq was used for library construction and sequencing (Sun et al., 2013). Genomic DNA was digested with HaeIII and RsaI, followed by sequencing adapter ligation, PCR amplification, and SLAF tag library construction. Paired-end sequencing was performed on the Illumina NovaSeq 6000 platform.

#### SNP discovery and quality control

After quality control of the sequencing data, reads were aligned to the onion reference genome (Allium cepa v2.0) using BWA. SNP calling was performed with GATK and SAMtools, with filtering criteria of missing rate ≤10%, MAF ≥0.05, and minimum sequencing depth ≥5×. High-quality SNP loci were retained for subsequent analysis. Among these, approximately 355,000 polymorphic loci were used for population structure and genetic diversity analyses.

#### Population structure analysis

ADMIXTURE (Alexander et al., 2009) was used for population structure analysis based on the full polymorphic SNP dataset, with the number of subpopulations (K) set from 1 to 10. The optimal K value was determined by cross-validation error. Principal component analysis (PCA) was performed using GCTA (Yang et al., 2011), and two-dimensional scatter plots were generated. A maximum likelihood (ML) phylogenetic tree was constructed with bootstrap support estimated from 1,000 replicates and visualized using the ggtree R package (Yu et al., 2017). Before PCA, SNP loci in strong linkage disequilibrium (LD) were pruned using PLINK (a 50-SNP sliding window, step size of 5, and r² > 0.2), and PCA was then performed on the LD-pruned dataset using GCTA.

#### Genetic diversity parameter calculation

Genetic diversity parameters were calculated for each subpopulation based on the full polymorphic SNP dataset, including minor allele frequency (MAF), observed heterozygosity (Ho), expected heterozygosity (He), Nei’s gene diversity index, Shannon–Wiener information index, and polymorphic information content (PIC). Nei’s gene diversity for each locus was calculated as 1 − Σp_i_², where p_i_ is the frequency of the i-th allele; for biallelic loci this equals 2pq and is identical to expected heterozygosity (He) under Hardy–Weinberg equilibrium.

#### SNP fingerprint construction

Candidate SNP markers for fingerprint construction were selected based on the following criteria: (1) even distribution across the genome; (2) locus completeness of 100%; (3) MAF ≥20%; (4) PIC ≥0.35; (5) Hardy–Weinberg equilibrium (HWE) test *p*-value > 0.01; and (6) no other mutations within 100 bp flanking the marker. A total of 335 polymorphic loci were identified as candidate markers for fingerprint construction of the 87 accessions. The detailed information of these markers (marker ID, chromosome, physical position, alleles, PIC value, and minor allele frequency) and the genotype calls of all 87 accessions at these loci are provided in **Supplementary Table S1**. Because these markers were deliberately selected for their high polymorphism and discriminatory power (MAF ≥20%, PIC ≥0.35), their observed heterozygosity (Ho) is higher than that of the genome-wide SNP set. Some markers exhibit an elevated Ho that is attributable to multi-copy or paralogous sequences captured by SLAF-seq rather than to genuine biallelic heterozygosity; this elevated heterozygosity does not affect the ability of these markers to uniquely distinguish the 87 accessions, and the diversity parameters reported for the fingerprint markers therefore do not represent the genome-wide level of genetic diversity of the population.

### Core collection construction and evaluation

#### Phenotype-based core collection construction

Based on the 29 agronomic traits, a core collection was screened using the QGA (Quantitative Genetic Analysis) method, combining Euclidean distance, random sampling, and shortest distance clustering at a sampling proportion of 25%.

#### SNP marker-based core collection construction

Based on the SNP marker data, the Core Hunter algorithm (Thachuk et al., 2009) was used to screen a core collection at approximately 30% sampling proportion. Core Hunter integrates multiple genetic measures—including allelic diversity, Shannon–Wiener index, and genetic distance—to maximize the genetic representativeness of the core collection.

#### Integrated core collection screening and evaluation

The phenotypic and SNP-based core collections were intersected to obtain the final core collection. Representativeness was evaluated using: (1) mean difference percentage (MD%); (2) range coincidence rate (CR%); and (3) coefficient of variation change rate (VR%). PCA was also used to compare the coverage of genetic variation between the core collection and the original population. To quantify the percentage of genetic variation space covered, the area of the convex hull enclosing the nine core accessions was divided by that enclosing all 87 accessions, using the first two principal components of the SNP-based PCA; convex hulls were computed with the “geometry” R package (convhulln function).

## Results

### Phenotypic genetic diversity analysis

#### Trait variation and genetic diversity

Genetic diversity was assessed for 29 agronomic traits across 87 accessions (**Table 2**). The coefficient of variation (CV) ranged from 14.27% to 65.41%, and the genetic diversity index (GDI) from 0.514 to 2.234. Among quantitative traits, pseudostem height showed the highest CV (65.41%) and flesh scale thickness the lowest (22.62%). Among qualitative traits, plant morphology had the highest CV (29.73%) and pseudostem skin color the lowest (14.27%). The trait with the highest GDI was pseudostem diameter (2.234), and the lowest was pseudostem skin color (0.514). These results indicate moderate to high phenotypic variation across the tested accessions.

**Table 2:** Variation and genetic diversity analysis of 29 agronomic traits.

Trait	Mean	Max	Min	SD	CV (%)	GDI
Plant height (cm)	21.91	32.09	7.00	5.06	23.10	2.187
Leaf spread (cm)	22.54	113.09	3.40	13.57	60.19	2.056
Tube leaf length (cm)	25.44	40.45	6.75	6.74	26.50	2.213
Tube leaf transverse diameter (mm)	4.07	7.06	1.66	1.05	25.68	2.189
Tube leaf number	27.82	53.43	3.00	13.35	48.00	2.104
Pseudostem height (mm)	3.30	11.99	0.20	2.16	65.41	2.156
Pseudostem diameter (mm)	5.53	10.14	2.25	1.31	23.65	2.234
Tillering number	6.40	13.00	1.33	2.64	41.34	2.178
Bulb weight (kg)	0.03	0.10	0.01	0.02	50.05	2.201
Disc stem diameter (mm)	8.62	29.89	3.45	2.76	32.02	2.167
Bulb longitudinal diameter (mm)	31.97	53.21	8.06	7.73	24.19	2.198
Bulb transverse diameter (mm)	32.16	53.21	7.81	8.87	27.58	2.224
Bulb neck diameter (mm)	8.45	15.14	1.92	2.66	31.45	2.231
Membranous scale leaf number	2.83	4.60	1.00	0.73	25.74	2.056
Flesh scale stripe number	23.06	38.07	10.00	4.63	20.06	2.178
Yield per plant (kg)	0.14	0.44	0.01	0.09	62.56	2.201
Flesh scale number	4.45	12.20	3.00	1.78	39.97	1.987
Flesh scale thickness (mm)	3.05	4.87	1.58	0.69	22.62	2.213
Bulblet number	2.84	5.00	1.00	0.88	31.18	2.134
Leaf color	2.08	3.00	1.00	0.51	24.55	0.748
Leaf waxiness	1.97	3.00	1.00	0.56	28.45	0.822
Plant morphology	2.28	3.00	1.00	0.68	29.73	1.036
Pseudostem skin color	2.86	3.00	1.00	0.41	14.27	0.514
Scale leaf color	2.20	3.00	1.00	0.55	24.89	0.807
Total flavonoid yield (mg/g)	3.93	5.72	1.88	0.92	23.31	2.187
Dry matter content (%)	17.60	30.69	3.44	4.10	23.30	2.201
Soluble solids (%)	19.57	31.81	13.01	6.40	32.70	2.134
Soluble sugar (%)	9.73	17.29	4.54	2.90	29.80	2.213
Soluble protein (mg/g FW)	6.07	10.40	1.25	1.67	27.47	2.201

#### Correlation analysis

Pearson correlation analysis of the 29 traits revealed 137 significantly correlated trait pairs (*p* < 0.05), of which 100 were significant at *p* < 0.01 (**Figure 1**). Tillering number was positively correlated with yield per plant (*r* = 0.68). Bulb longitudinal diameter was strongly correlated with bulb transverse diameter (*r* = 0.72), and pseudostem diameter with bulb transverse diameter (*r* = 0.65). Plant height correlated with tube leaf length (*r* = 0.61), and pseudostem diameter with bulb weight (*r* = 0.58). These coordinated variation patterns provided a basis for identifying representative traits.

**Figure 1:**
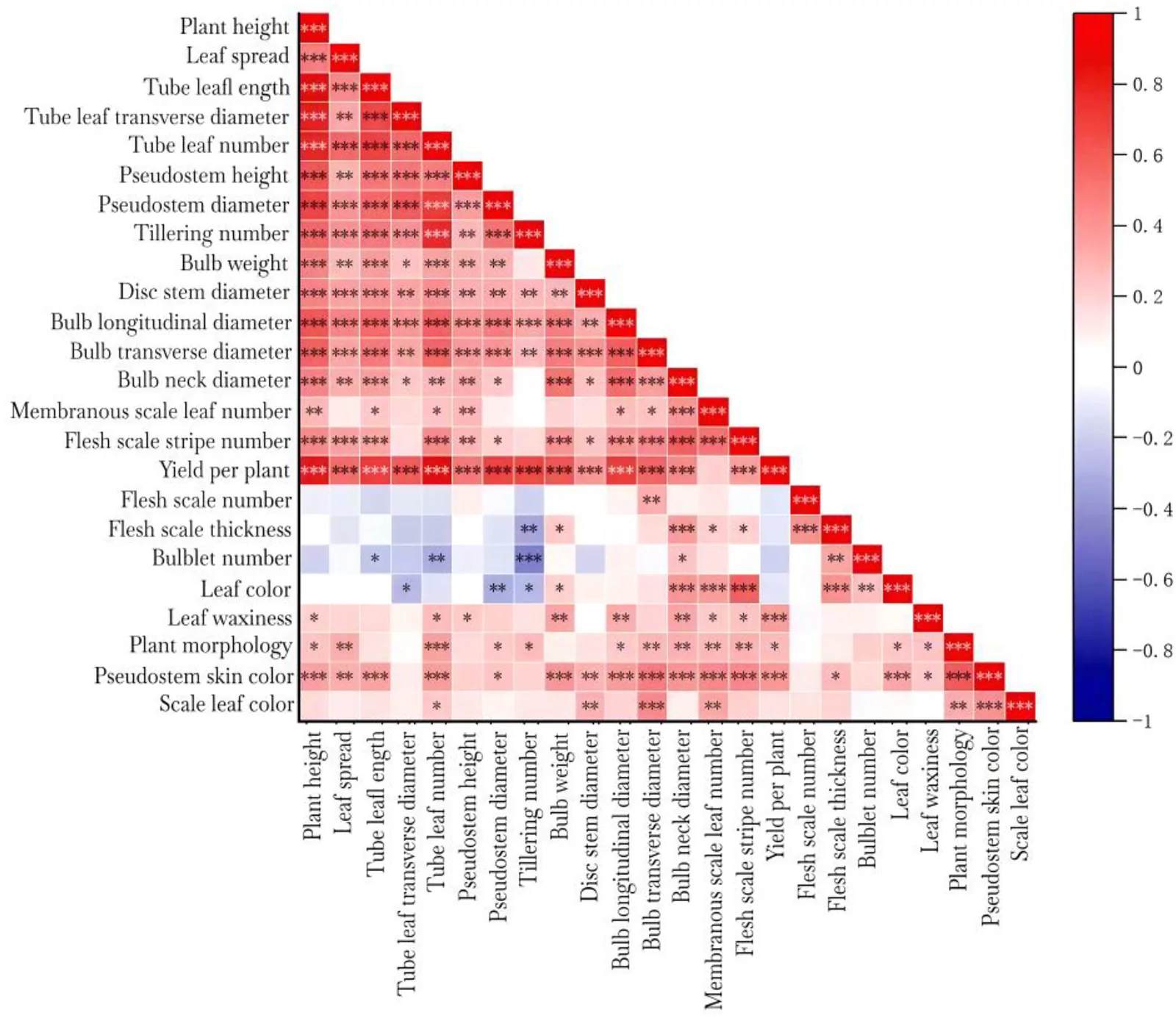
Correlation analysis of various traits in tillering onion. *p <=0.05, **p <=0.01, ***p <=0.001.

#### Principal component analysis

Principal component analysis of the 29 traits yielded 10 components with eigenvalues ≥0.8 and a cumulative contribution rate of 79.312% (**Table 3**). PC1 had the highest eigenvalue (9.181) and contributed 31.657% of the variance, primarily reflecting bulb size and plant growth vigor. PC2 had an eigenvalue of 3.732 and a contribution rate of 12.870%. The first five components accounted for 62.469% of cumulative variance, covering plant type, bulb characteristics, yield, and quality.

**Table 3:** Principal component analysis of 29 agronomic traits (total variance explained).

Component	Eigenvalue	Variance (%)	Cumulative (%)
PC1	9.181	31.657	31.657
PC2	3.732	12.870	44.527
PC3	2.092	7.213	51.740
PC4	1.645	5.674	57.414
PC5	1.466	5.055	62.469
PC6	1.126	3.881	66.350
PC7	1.083	3.733	70.083
PC8	0.953	3.286	73.369
PC9	0.915	3.156	76.525
PC10	0.808	2.787	79.312

Factor scores from the 10 components were used to calculate a composite score (F-value) for each accession. The F-value was highly correlated with 22 traits (*p* < 0.01), positively correlated with bulblet number (*p* < 0.05), and negatively correlated with soluble solids content (*p* < 0.05) (**Table 4**). Plant height (*r* = 0.827**), tube leaf length (*r* = 0.731**), yield per plant (*r* = 0.715**), bulb transverse diameter (*r* = 0.712**), and bulb longitudinal diameter (*r* = 0.690**) showed the strongest correlations with the F-value, identifying these as key indicators for evaluating comprehensive performance.

**Table 4:** Correlation coefficients between composite value (F-value) and 29 agronomic traits.

Trait	Correlation coefficient	Trait	Correlation coefficient
Plant height	0.827**	Flesh scale stripe number	0.611**
Leaf spread	0.495**	Yield per plant	0.715**
Tube leaf length	0.731**	Flesh scale thickness	0.295**
Tube leaf transverse diameter	0.614**	Bulblet number	0.271*
Tube leaf number	0.644**	Leaf color	0.126
Pseudostem height	0.568**	Leaf waxiness	0.328**
Pseudostem diameter	0.646**	Plant morphology	0.439**
Tillering number	0.311**	Pseudostem skin color	0.618**
Bulb weight	0.571**	Scale leaf color	0.359**
Disc stem diameter	0.527**	Soluble protein	-0.122
Bulb longitudinal diameter	0.690**	Soluble solids	-0.220*
Bulb transverse diameter	0.712**	Soluble sugar	-0.406**
Bulb neck diameter	0.664**	Total flavonoid yield	0.167
Flesh scale number	0.097	Dry matter content	-0.041
Membranous scale leaf number	0.532**		

*indicates significance at the 0.05 level; **indicates significance at the 0.01 level.

Stepwise linear regression, using the 29 traits as independent variables and the F-value as the dependent variable, identified 14 typical traits (R² = 0.971, *p* < 0.01): plant height, pseudostem height, pseudostem diameter, tillering number, disc stem diameter, bulb longitudinal diameter, bulb transverse diameter, bulb neck diameter, membranous scale leaf number, flesh scale thickness, plant morphology, pseudostem skin color, scale leaf color, and soluble solids (**Table 5**). These 14 traits explained 97.1% of the variation in the F-value, effectively reducing redundancy among traits. Notably, although tillering number showed a positive bivariate correlation with the F-value (r = 0.311, p < 0.01; **Table 4**), its standardized coefficient in the stepwise model was negative (−0.163; **Table 5**). This sign reversal reflects multicollinearity and a suppression effect among highly correlated yield components (e.g., bulb weight, bulb diameters, and yield per plant), such that the coefficient represents the conditional effect of tillering number after controlling for these correlated traits. All variance inflation factors (VIF) in the final model were below 5, indicating that multicollinearity, although present, was not severe.

**Table 5:** Stepwise linear regression of composite value (F-value) and 29 agronomic traits.

Trait	Regression coefficient	95% CI	VIF	Tolerance
Constant	-1.998** (-32.559)	-2.118 ~ -1.877	–	–
Plant height	0.921** (9.466)	0.731 ~ 1.112	4.501	0.222
Pseudostem height	0.278** (3.715)	0.131 ~ 0.424	2.188	0.457
Pseudostem diameter	0.465** (6.254)	0.319 ~ 0.611	1.774	0.564
Tillering number	-0.163** (-2.776)	-0.279 ~ -0.048	2.078	0.481
Disc stem diameter	0.456** (4.293)	0.248 ~ 0.664	1.435	0.697
Bulb longitudinal diameter	0.246** (2.986)	0.085 ~ 0.408	2.328	0.429
Bulb transverse diameter	0.428** (6.022)	0.289 ~ 0.567	2.248	0.445
Bulb neck diameter	0.148* (2.085)	0.009 ~ 0.287	2.379	0.421
Membranous scale leaf number	0.254** (4.440)	0.142 ~ 0.365	1.561	0.641
Flesh scale thickness	0.151** (2.726)	0.043 ~ 0.260	1.583	0.632
Plant morphology	0.131** (3.603)	0.060 ~ 0.203	1.779	0.562
Pseudostem skin color	0.328** (4.653)	0.190 ~ 0.466	2.417	0.414
Scale leaf color	0.113* (2.596)	0.028 ~ 0.198	1.642	0.609
Soluble solids	0.228** (5.958)	0.153 ~ 0.302	1.667	0.600
Sample size	87			
R²	0.971			
Adjusted R²	0.965			
F-value	F(14, 72) = 171.841, p < 0.001			

*indicates significance at the 0.05 level; **indicates significance at the 0.01 level. Values in parentheses are t-statistics.

#### Cluster analysis

Based on the 14 typical traits, UPGMA clustering with squared Euclidean distance divided the 87 accessions into eight groups at a distance of 15 (**Figure 2**). The cophenetic correlation coefficient was 0.828, indicating good fidelity of the dendrogram to the underlying distance matrix, and the average silhouette width was 0.179. Although this silhouette width is low, it reflects the predominance of one large group (Group I) together with several biologically distinctive singleton clusters carrying extreme trait values, which were retained as biologically meaningful units.

**Figure 2:**
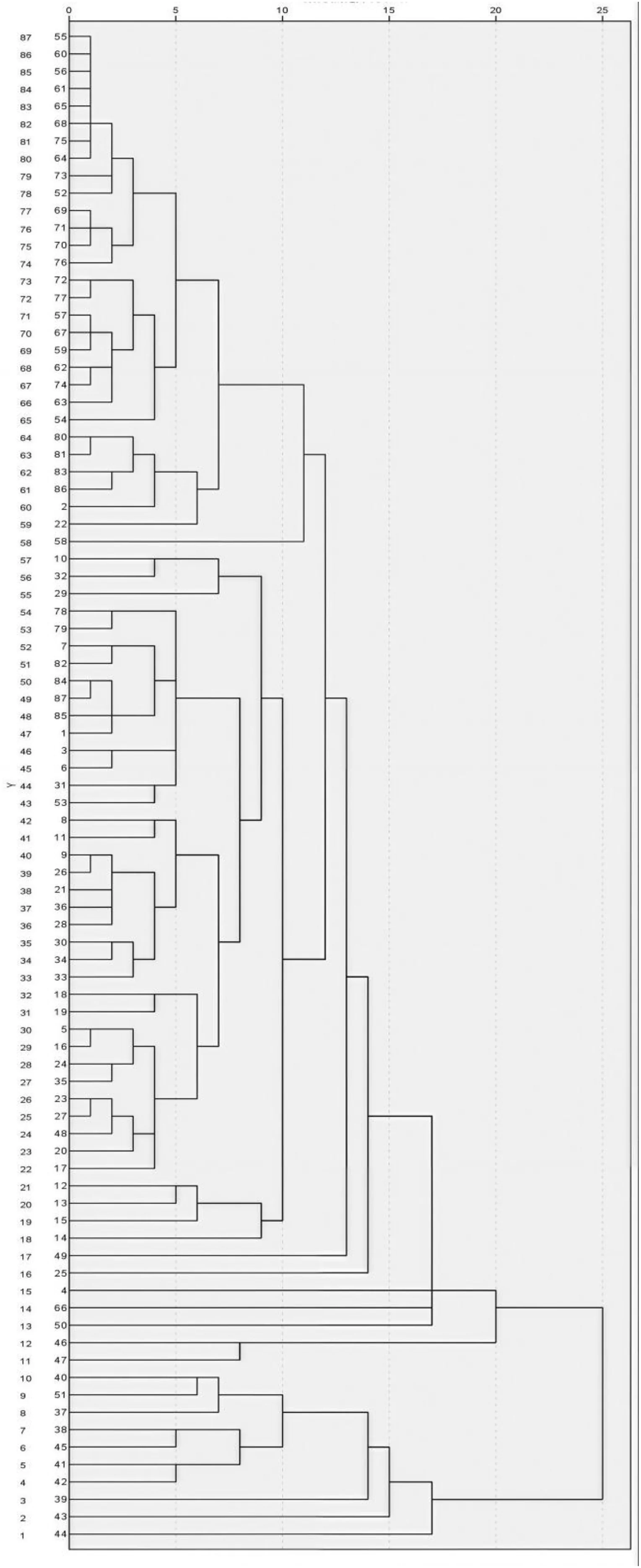
Dendrogram of cluster analysis for 87 tillering onion accessions based on 14 typical traits.

Group I contained 72 accessions (82.8% of the total), representing the main phenotypic profile of tillering onion. Among these, 41 were from Jilin, 19 from Heilongjiang, 7 from Liaoning, 3 from Shandong, and 2 from Hebei. All accessions in this group had purplish-red pseudostem skin, most had reddish-brown scale leaves, and plant morphology was predominantly upright. Quantitative traits such as plant height (21.91 cm), pseudostem height (3.03 mm), and mean soluble solids (20.02%) were at intermediate levels, serving as a baseline for comparison.

Group II contained only M4 (Heilongjiang), characterized by a markedly oblate bulb (longitudinal-to-transverse diameter ratio < 0.6) and the highest number of membranous scale leaf layers, indicating a compact bulb structure.

Group III contained only M66 (Jilin), an upright type with the largest bulb size—both longitudinal and transverse diameters were the maximum across all accessions, and the bulb neck was the thickest (14.95 mm).

Group IV contained only M50 (Heilongjiang), with the tallest pseudostem (11.99 mm) and a soluble solids content of 29.16%, representing a potential high-quality breeding material.

Group V contained two accessions, M46 and M47 (from Guangxi and Jilin, respectively), with a spreading growth habit, short stature (mean height 8.25 cm), low pseudostem (1.00 mm), and relatively low soluble solids (14.05%).

Group VI contained 8 accessions (M37, M38, M39, M40, M41, M42, M45, M51; 6 from Guangxi and 2 from Jilin). All had upright morphology, but scale leaf color varied between yellowish-white and reddish-brown—unlike Group I, where it was almost entirely reddish-brown—suggesting specific genetic variation within this group.

Group VII contained only M43 (Guangxi), with the narrowest tube leaves and the thickest pseudostem, a distinctive “narrow-leaf thick-stem” morphology.

Group VIII contained only M44 (Guangxi), with a mean tillering number of 13 per plant—far exceeding all other accessions—representing a distinctive germplasm with high reproductive capacity.

The clustering revealed a geographic pattern: Group I spanned five provinces, Group V covered two provinces, and Groups II, III, IV, VI, VII, and VIII each originated from a single province. The results suggest that phenotypic variation in tillering onion reflects both geographic clustering and cross-regional environmental influences.

### SNP marker genetic diversity analysis

#### Sequencing quality and SLAF tag statistics

SLAF-seq of the 87 accessions yielded 663,604 SLAF tags with an average sequencing depth of 12.01×. The tags were evenly distributed across the genome with appropriate density, confirming good library quality. After SNP calling and filtering, 1,209,763 high-quality SNP loci were retained for analysis. The density distribution of SNP loci across chromosomes is shown in **Figure 3**.

**Figure 3:**
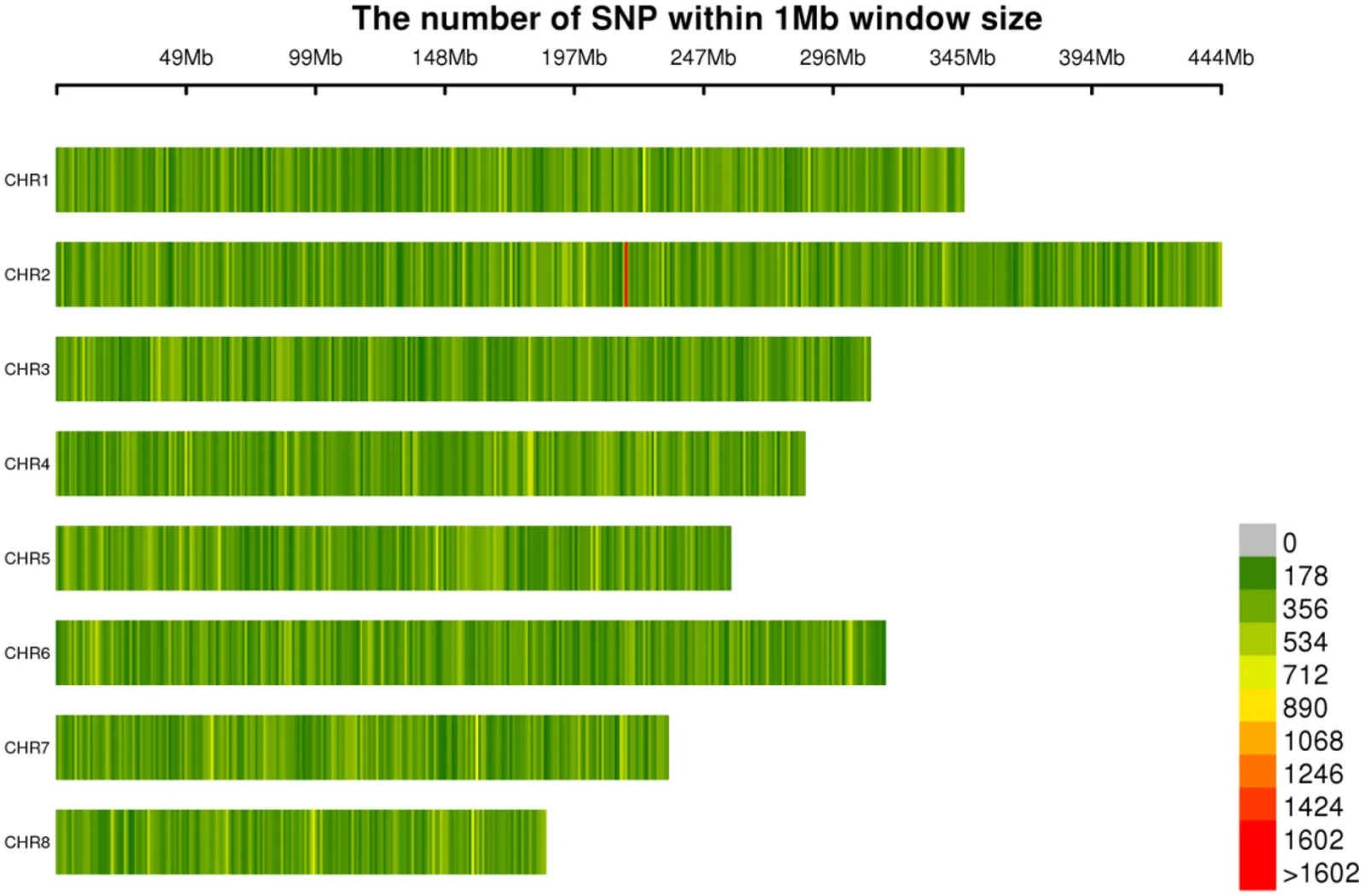
Distribution density of SNP loci on chromosomes.

#### Population structure analysis

ADMIXTURE analysis identified K = 2 as optimal based on the lowest cross-validation error (**Figure 4**, **5**), dividing the 87 accessions into two subpopulations (C1, C2). C1 contained 10 accessions, including all 9 from Guangxi (M37–M40, M42–M46) plus M49 (Heilongjiang); C2 contained the remaining 77 accessions from Jilin, Heilongjiang, Liaoning, Shandong, and Hebei. The subpopulation division largely corresponded to a south–north geographic pattern, with C1 dominated by southern (Guangxi) accessions, although one accession (M49, Heilongjiang) crossed this boundary, indicating some gene flow among accessions.

**Figure 4:**
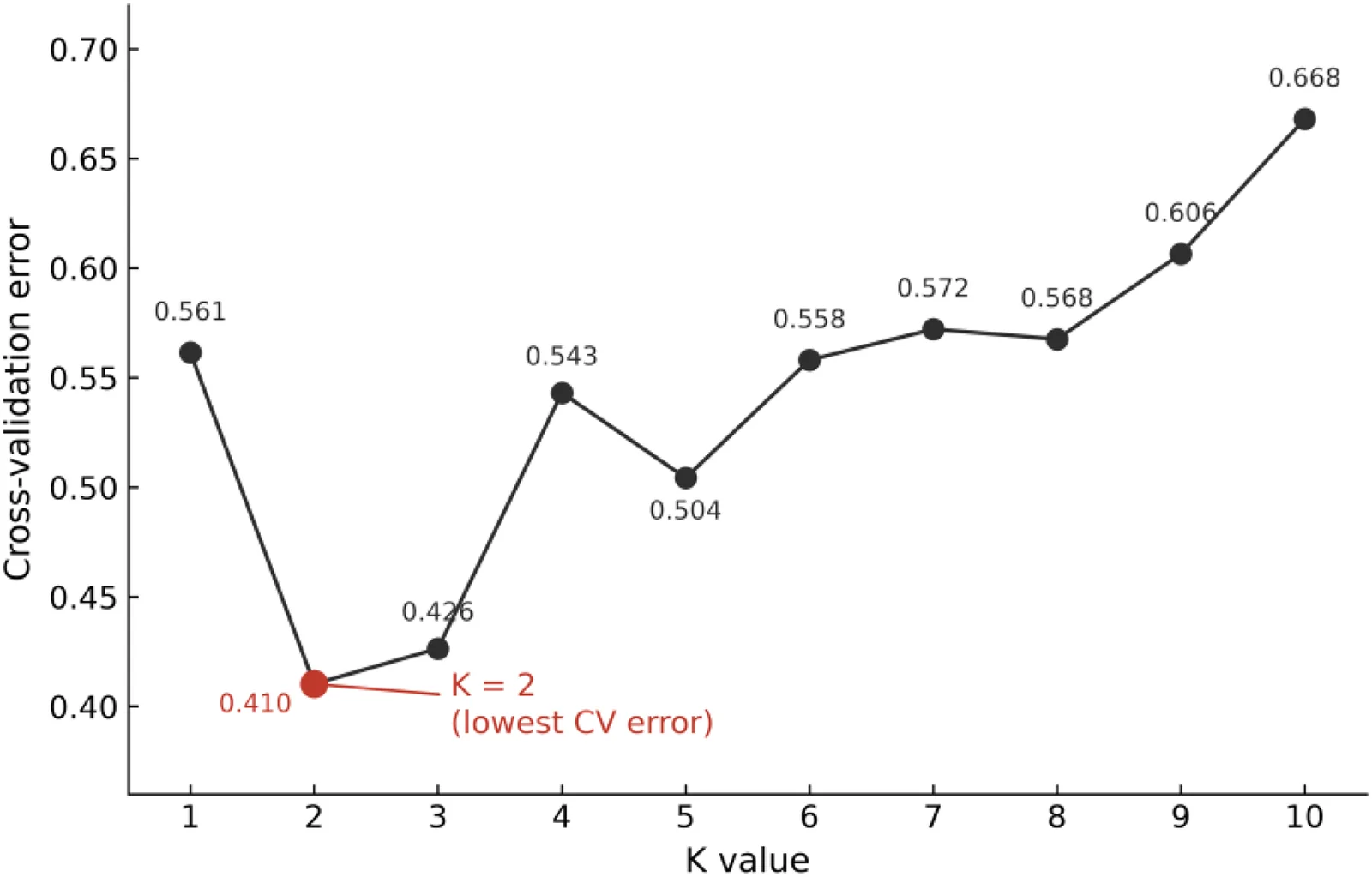
Cross-validation error of different K values in population structure analysis. Cross-validation error of different K values in population structure analysis based on the full polymorphic SNP dataset. The cross-validation error values were 0.56147 (K = 1), 0.41023 (K = 2), 0.42641 (K = 3), 0.54310 (K = 4), 0.50432 (K = 5), 0.55802 (K = 6), 0.57212 (K = 7), 0.56760 (K = 8), 0.60649 (K = 9), and 0.66812 (K = 10); the lowest value occurred at K = 2.

**Figure 5:**
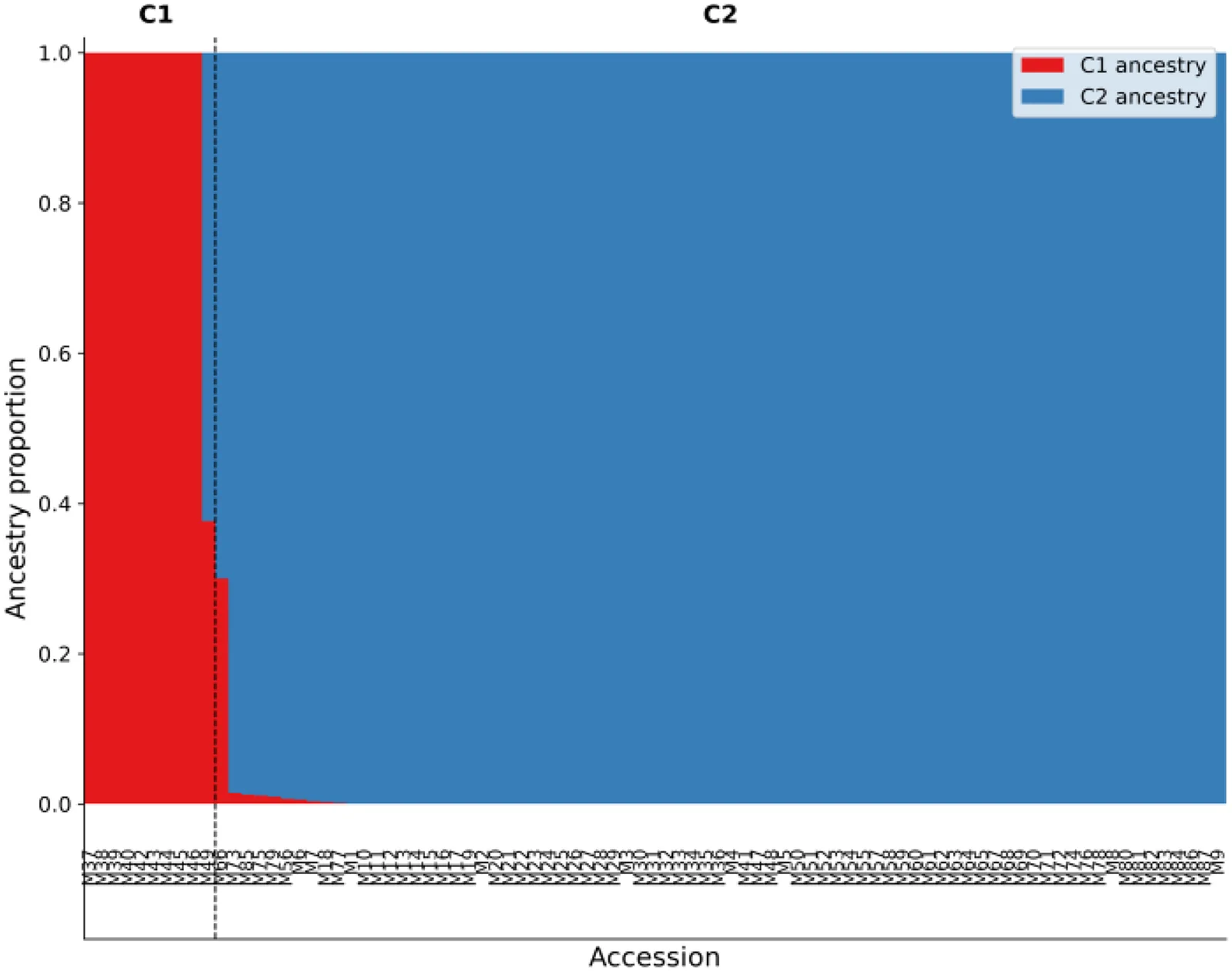
Population structure of 87 tillering onion accessions at K = 2 (ADMIXTURE analysis).

#### Principal component analysis

PCA based on SNP data showed that PC1, PC2, and PC3 explained 40.59%, 6.28%, and 2.66% of the total genetic variation, respectively (**Figure 6**). On the PC1–PC2 plane, the 87 accessions formed two clusters consistent with the ADMIXTURE results. The two clusters were largely separated along PC1, consistent with the south–north divergence revealed by ADMIXTURE.

**Figure 6:**
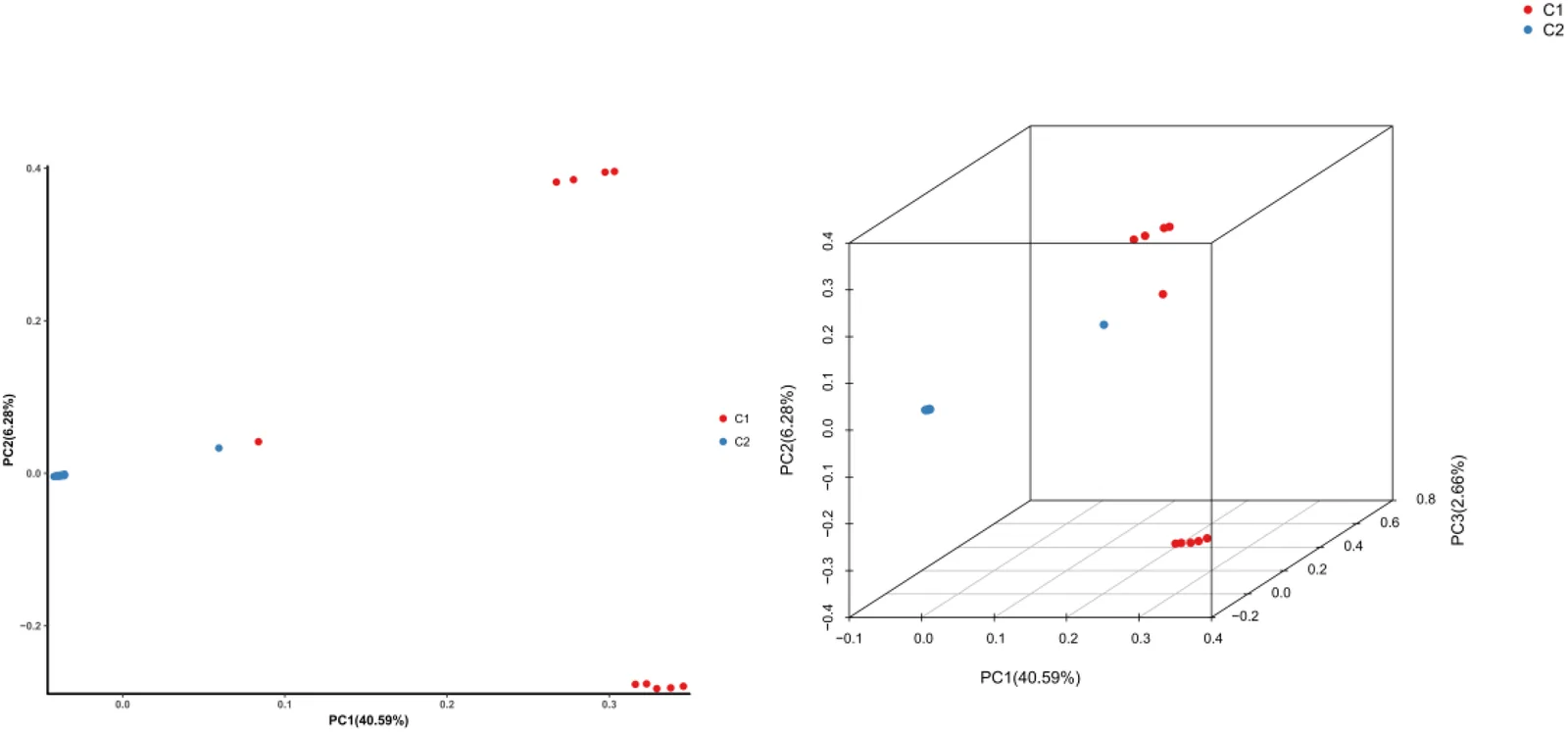
PCA of 87 tillering onion accessions based on SNP markers, shown as a two-dimensional (PC1 vs. PC2) and a three-dimensional (PC1–PC3) projection.

#### Genetic diversity parameter analysis

Genetic diversity parameters for the two subpopulations are shown in **Table 6**. C1 had 295,429 polymorphic markers (83.2% of the total loci), with MAF of 0.216, Nei’s index of 0.281, PIC of 0.224, and Shannon–Wiener index of 0.422. C2 had 257,488 polymorphic markers (72.5%), with MAF of 0.238, Nei’s index of 0.274, PIC of 0.212, and Shannon–Wiener index of 0.396. Although C1 contained far fewer accessions (10 vs. 77), its diversity parameters were slightly higher, reflecting the distinct genetic background of the Guangxi accessions.

**Table 6:** Genetic diversity parameters of two subpopulations.

Subpopulation	No. of polymorphic markers	MAF	Nei’s index	PIC	Shannon–Wiener index
C1	295,429	0.216	0.281	0.224	0.422
C2	257,488	0.238	0.274	0.212	0.396

#### Phylogenetic analysis

A maximum likelihood (ML) tree showed that the 87 accessions formed multiple distinct branches (**Figure 7**), broadly consistent with the population structure and PCA results. Accessions from the same region tended to cluster together, but cross-regional clustering was also observed. All 10 accessions of the C1 subpopulation (nine from Guangxi: M37–M40 and M42–M46, plus M49 from Heilongjiang) formed a relatively independent branch, suggesting a unique genetic background.

**Figure 7:**
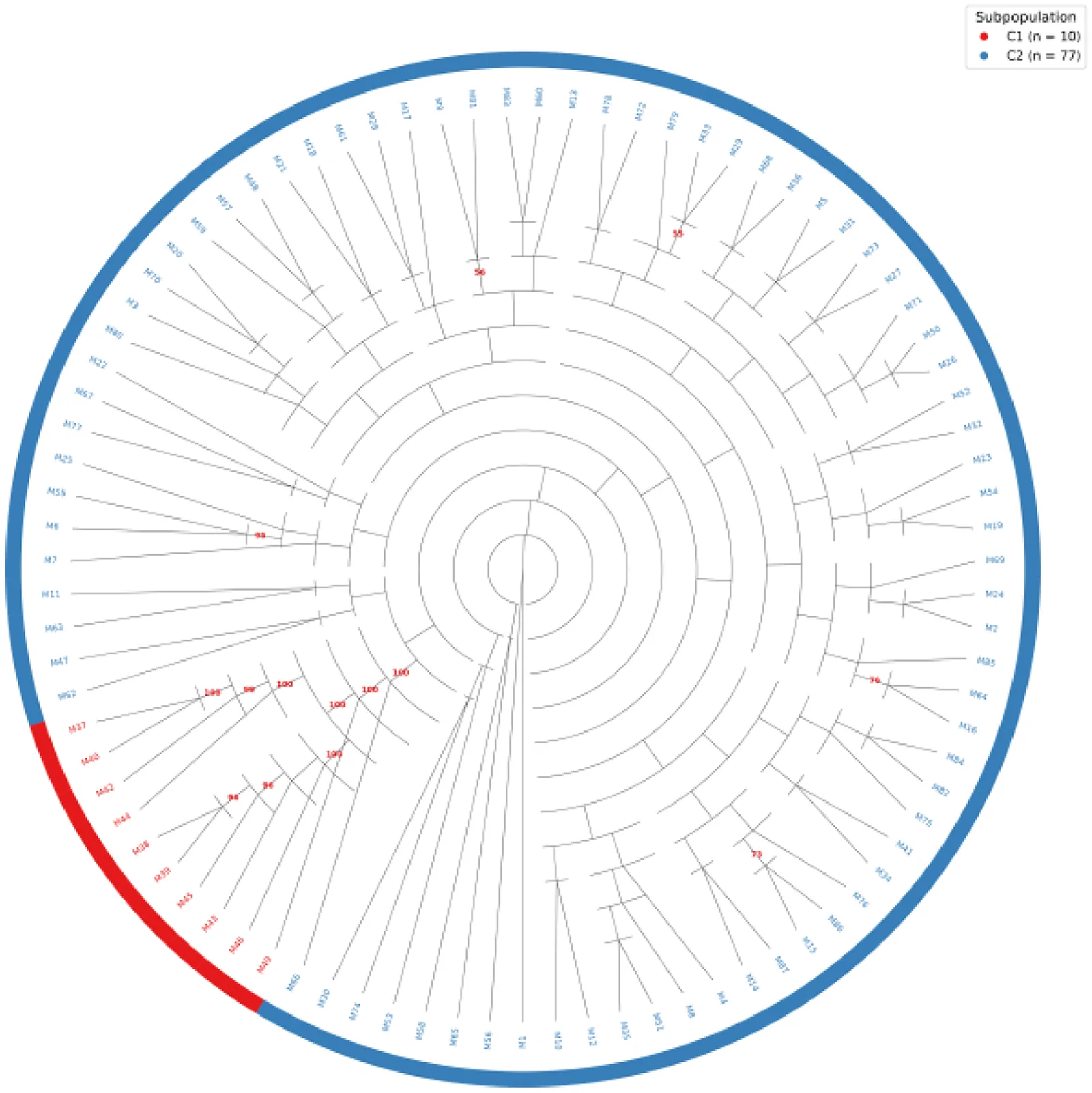
Maximum likelihood (ML) phylogenetic tree of 87 tillering onion accessions based on SNP markers. The colored ring indicates the subpopulation assignment (C1, C2). Node labels indicate bootstrap support values based on 1,000 replicates.

#### SNP fingerprint construction and genetic diversity evaluation

From 1,209,763 SNP loci, 335 high-quality candidate markers were selected for fingerprint construction based on the six stringent criteria described in the Methods (**Supplementary Table S1**). Across the 87 accessions at these 335 loci (**Table 7**), observed heterozygosity (Ho) ranged from 0.379 to 0.644 (mean 0.572), expected heterozygosity (He) from 0.455 to 0.500 (mean 0.479), Nei’s diversity index from 0.455 to 0.500 (mean 0.479), Shannon–Wiener index from 0.648 to 0.693 (mean 0.672), and PIC from 0.352 to 0.375 (mean 0.364). The relatively high mean Ho (0.572) reflects the deliberate selection of these markers for high polymorphism and, in part, the capture of multi-copy or paralogous sequences by SLAF-seq. Because these markers were chosen for their discriminatory power (PIC), this elevated heterozygosity does not compromise their ability to uniquely distinguish all 87 accessions, and the diversity parameters of the fingerprint set should not be interpreted as the genome-wide diversity level of the population. The distribution of genetic diversity parameters is shown in **Figure 8**.

**Table 7:** Genetic diversity evaluation of the whole population and SNP core collection. Values are based on the 335 fingerprint markers selected for the 87 accessions.

Population	No.	Ho	He	Nei’s index	Shannon–Wiener index	PIC
All	87	0.379–0.644 (0.572)	0.455–0.500 (0.479)	0.455–0.500 (0.479)	0.648–0.693 (0.672)	0.352–0.375 (0.364)
SNP Core	26	0.154–0.654 (0.372)	0.311–0.500 (0.472)	0.311–0.500 (0.472)	0.490–0.693 (0.664)	0.262–0.375 (0.360)

Values in parentheses are means.

**Figure 8:**
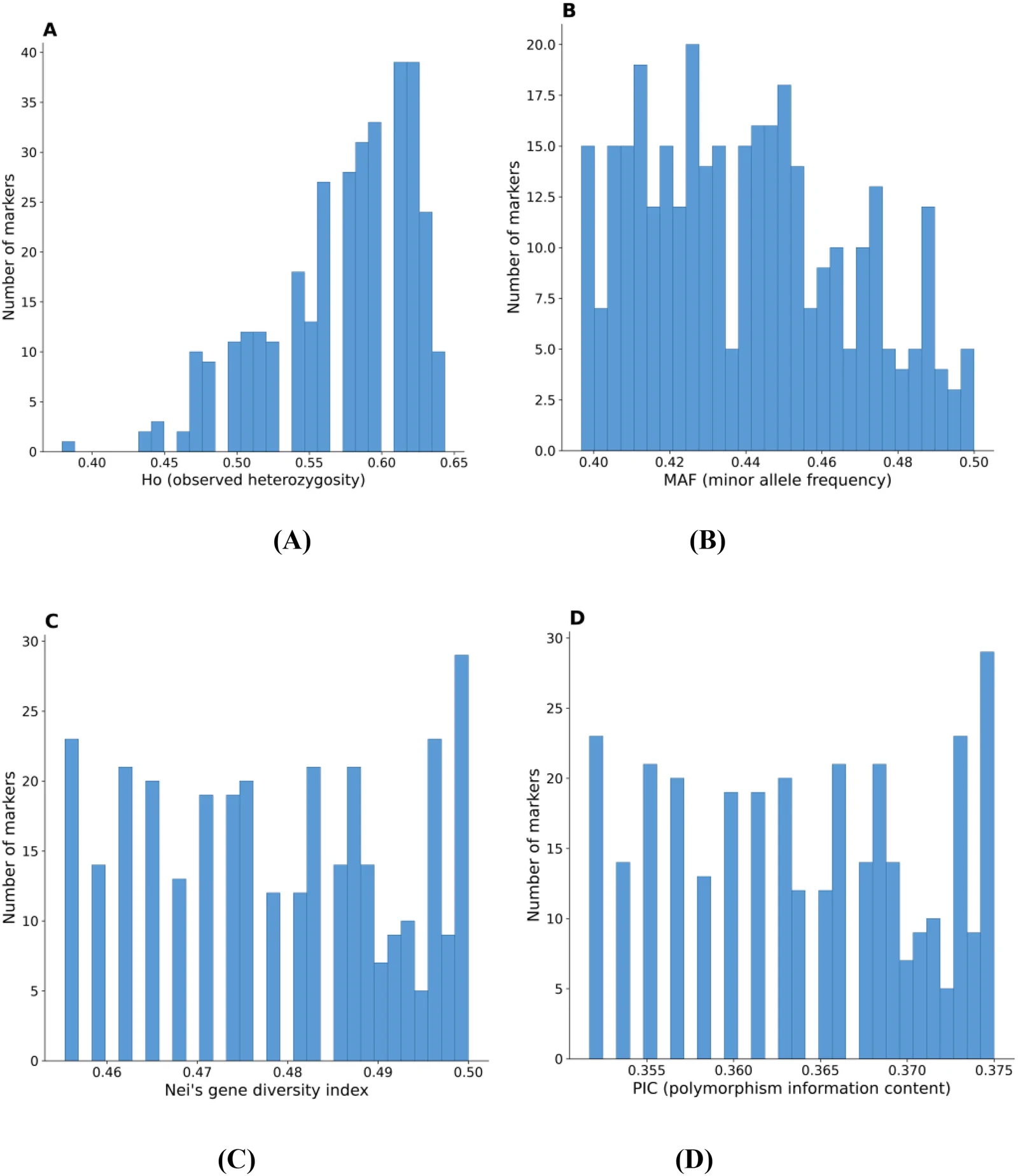
Distribution of genetic diversity parameters [**(A)** Ho; **(B)** MAF; **(C)** Nei’s index; **(D)** PIC].

### Core collection construction and evaluation

#### Phenotype-based core collection construction

Using the QGA method at 25% sampling proportion, 21 phenotypic core accessions were selected from the 87 accessions. These included representative materials from all eight cluster groups, and the mean, range, and coefficient of variation of each trait were generally consistent with those of the original population.

#### SNP marker-based core collection construction

Using Core Hunter at approximately 30% sampling proportion, 26 SNP core accessions were selected. Genetic diversity evaluation of this SNP core (26 accessions; **Table 7**) showed that the mean Nei’s index (0.472), Shannon–Wiener index (0.664), and PIC (0.360) were close to those of the whole population (0.479, 0.672, and 0.364, respectively), indicating effective capture of genetic variation at the molecular level.

#### Integrated core collection screening and evaluation

Intersecting the phenotypic core (21 accessions) and the SNP-based core (26 accessions) yielded a final core collection of 9 accessions: M15, M24, M28, M37, M45, M47, M49, M62, and M66, representing 10.34% of the total resources. These nine accessions originated from Jilin (M15, M24, M45, M47, M62, M66), Liaoning (M28), Guangxi (M37), and Heilongjiang (M49), covering both subpopulations (three from C1 and six from C2). Phenotypically, these nine accessions represented four of the eight cluster groups (Groups I, III, V, and VI), encompassing the dominant main phenotype and the distinctive large-bulb, spreading-dwarf, and scale-leaf-color-variant types.

Evaluation parameters were: MD% = 10.0% (< 20%), CR% = 83.6% (> 80%), and VR% = 112.2% (> 100%), all meeting the standards for core collection construction. For comparison, an alternative core collection of the same size (nine accessions) constructed by Core Hunter with joint optimization on both phenotypic and genotypic data showed MD% = 13.7%, CR% = 79.9%, VR% = 146.3%, and an allele retention rate of 98.05%; the intersection core performed comparably or better on these parameters, indicating that it was not inferior to the jointly optimized core of the same size. PCA comparing the core collection with the whole population (**Figure 9**) showed that the nine core accessions covered approximately 87.3% of the genetic variation space of the original population. In **Figure 9** the axes are labelled with the variance explained by each principal component, and all 87 accessions are shown together with the nine core accessions. At the phenotypic level, the nine core accessions preserved the major range of the 24 quantitative traits, with a coincidence rate of range (CR%) of 83.6%; notably, the key yield-related traits bulb weight and yield per plant were retained at over 95% of their full ranges. In addition, the nine core accessions retained 98.38% of all alleles and 92.43% of low-frequency alleles (MAF < 0.1), with a mean Nei’s genetic distance of 0.321 among them and observed and expected heterozygosity of 0.355 and 0.361, respectively, further confirming that the core collection captures the genetic variation of the whole population.

**Figure 9:**
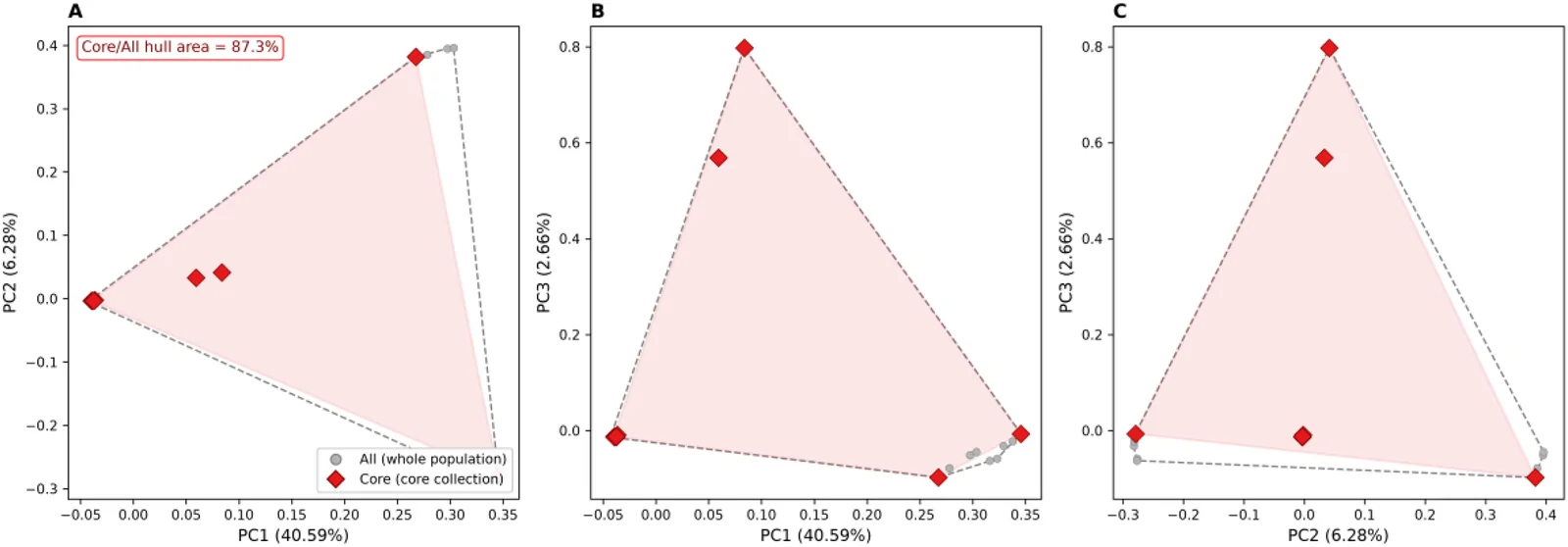
PCA comparison between the nine-accession core collection and the whole population (87 accessions) based on SNP markers. Three panels are shown: **(A)** PC1 vs. PC2, **(B)** PC1 vs. PC3, and **(C)** PC2 vs. PC3. Axis labels include the percentage of variance explained by each principal component. Gray circles indicate all 87 accessions, and red diamonds indicate the nine core accessions. Dashed gray and red polygons denote the convex hulls of the whole population and the core collection, respectively.

## Discussion

### Phenotypic genetic diversity

The 29 agronomic traits across 87 accessions showed a CV range of 14.27%–65.41% and GDI range of 0.514–2.234, consistent with the findings of Li et al. (2023) for 69 tillering onion accessions. Pseudostem height had the highest CV (65.41%), indicating substantial variation and potential for genetic improvement, while pseudostem skin color was the most stable trait (CV = 14.27%). The positive correlation between tillering number and yield per plant (*r* = 0.68) aligns with the fact that tillering is the primary production target of this crop. PCA extracted 10 components (79.31% cumulative variance), and stepwise regression identified 14 representative traits (R² = 0.971) spanning plant morphology, yield components, and quality—reducing redundancy and providing a reliable basis for core collection construction. The negative standardized coefficient of tillering number in the stepwise model, despite its positive bivariate correlation with the composite F-value, is attributable to multicollinearity and a suppression effect among yield-related traits, highlighting that regression coefficients should be interpreted as conditional effects rather than simple causal influences. It should be noted that the phenotypic data were collected from a single environment and a single growing season (Jilin, 2024); the estimated trait variation and the phenotypic core collection may therefore be influenced by genotype × environment interactions, and multi-environment evaluation would be needed to confirm their stability. Leave-one-out cross-validation of the stepwise model yielded a cross-validated R² of 0.955, confirming the stability of the 14 representative traits in recovering the composite F-value. Because the composite F-value is derived from the same 29 traits, the stepwise model is interpreted as a description of the composition of the composite score rather than as an independent predictive test.

Cluster analysis divided the accessions into eight groups, with Group I (72 accessions, 82.8%) as the largest, spanning five provinces and representing the typical phenotype. The remaining seven groups each showed distinct features: oblate bulbs (M4), large bulbs (M66), tall pseudostem with high soluble solids (M50), spreading dwarf habit (M46, M47), scale leaf color variation (Group VI), narrow-leaf thick-stem morphology (M43), and high tillering capacity (M44, 13 tillers per plant). The broad distribution of Group I and the single-province origins of the other groups suggest that tillering onion germplasm contains both a widely adapted “main type” and geographically specific “special types.” These findings extend those of Jin et al. (2013), who analyzed 48 accessions with ISSR markers, and highlight the importance of retaining distinct groups in core collection construction to preserve elite alleles.

### SNP marker genetic diversity

SLAF-seq yielded 1,209,763 SNP loci across 87 accessions, far surpassing the polymorphism detection capacity of traditional SSR markers. Population structure analysis identified two subpopulations, with C1 (mainly Guangxi accessions) showing slightly higher diversity (Nei’s index 0.281, PIC 0.224) than C2 (Nei’s index 0.274, PIC 0.212), suggesting a distinct genetic background for the Guangxi accessions that warrants attention in breeding. The mean PIC of the whole population based on the 335 SNP dataset (0.364) was higher than that reported for garlic (0.31; Yang et al., 2026), although the higher mean PIC of the 335 fingerprint markers (0.364) reflects their deliberate selection for high polymorphism. Recent high-quality genomic resources for Allium crops, including the chromosome-level genome assemblies reported by Hao et al. (2023) and the genomic profiling of intraspecific divergence in Allium cepa by Packia Lekshmi et al. (2024), now provide a framework for interpreting these results and will facilitate functional characterization of the discriminating markers identified here.

The population structure, PCA, and phylogenetic analyses were mutually consistent in revealing genetic relationships among accessions. Notably, genetic relationships did not always match geographic origins—some accessions from different provinces clustered closely at the molecular level—likely reflecting introduction history and germplasm exchange.

### Core collection construction strategy

This study used a dual strategy: QGA based on phenotypic traits and Core Hunter based on SNP markers, with the final core obtained by intersection. This approach ensures representativeness at both the phenotypic and genomic levels while minimizing redundancy. The final nine core accessions (10.34% of the total) fall within the reasonable range after intersecting the recommended 20%–30% sampling proportion (Yonezawa et al., 1995). The evaluation parameters (MD% = 10.0%, CR% = 83.6%, VR% = 112.2%) met all criteria, and PCA confirmed effective coverage of the genetic variation space. In addition, a same-size core jointly optimized on both phenotypic and genotypic data (MD% = 13.7%, CR% = 79.9%, VR% = 146.3%, allele retention of 98.05%) showed no advantage over the intersection core, supporting the reliability of the dual-strategy intersection approach.

Compared with methods based solely on phenotypic or molecular data, this integrated strategy offers three advantages: (1) the phenotypic core ensures representativeness in agronomic traits; (2) the SNP core ensures coverage of genomic diversity; and (3) the intersection minimizes redundancy while maintaining representativeness. This strategy can serve as a reference for core collection construction in other crops. Nevertheless, this intersection strategy has a limitation worth noting: because the final core is defined as the intersection of the phenotypic and genotypic cores, it can exclude accessions that are individually important for either phenotypic or genotypic diversity, even when both core subsets perform well on their own. Accessions present in only one of the two subsets but carrying rare alleles or extreme trait values may therefore be lost. This trade-off should be weighed when applying the strategy to germplasm in which phenotypic and genotypic diversity are only partially correlated.

### Application value of SNP fingerprints

The 335 SNP markers used for fingerprint construction were selected under the six stringent criteria described in the Methods (even distribution across the genome, locus completeness 100%, MAF ≥20%, PIC ≥0.35, HWE p-value > 0.01, no other mutations within 100 bp flanking regions), ensuring reliability and discriminatory power. These fingerprints have potential applications in rapid germplasm identification, authenticity verification, cultivar-rights protection, hybrid-purity testing, and germplasm resource management, although these applications were not experimentally evaluated in the present study. Future work can combine the fingerprints with association analysis to identify markers linked to tillering number, yield, quality, and other traits, supporting marker-assisted selection in tillering onion breeding. The relatively high mean observed heterozygosity of the fingerprint markers (0.572) arises because SLAF-seq can capture multi-copy fragments rather than a single biallelic locus. This does not compromise the reliability or discriminatory power of the fingerprint, because these markers were selected for their PIC and can uniquely distinguish all 87 accessions. It should also be emphasized that these markers represent a deliberately high-polymorphism fingerprint panel, and their diversity parameters therefore do not reflect the genome-wide level of genetic diversity of tillering onion.

## Conclusion

This study systematically evaluated the genetic diversity of 87 tillering onion accessions using phenotypic traits and SNP markers. The main findings are:

1. The 29 agronomic traits showed a CV range of 14.27%–65.41% and GDI range of 0.514–2.234. PCA extracted 10 components (79.31% cumulative variance), and stepwise regression identified 14 representative traits (R² = 0.971). Cluster analysis classified the accessions into eight groups, confirming moderate to high phenotypic diversity.
2. SLAF-seq developed 1,209,763 SNP loci. Population structure analysis identified two subpopulations, with C1 showing slightly higher diversity. The 335-marker fingerprint revealed a mean Nei’s index of 0.479 and mean PIC of 0.364 across the population. PCA and phylogenetic analyses provided molecular evidence for the classification and utilization of tillering onion germplasm.
3. Integrating QGA and Core Hunter methods yielded a core collection of nine accessions (M15, M24, M28, M37, M45, M47, M49, M62, M66; 10.34% of the total). Evaluation parameters (MD% = 10.0%, CR% = 83.6%, VR% = 112.2%) and PCA verification confirmed good representativeness.

These results provide a foundation for efficient germplasm conservation, elite accession identification, and parental selection in tillering onion breeding.

## Data Availability

The sequencing data have been deposited in the NCBI SRA under BioProject PRJNA1511634. The SNP genotype data of the 335 fingerprint markers for the 87 accessions are provided in **Supplementary Table S1**. All other data supporting the conclusions are available from the corresponding author upon reasonable request.
